# Prospective evaluation of Chondroitin sulfate, Heparan sulfate and Hyaluronic acid in prostate cancer

**DOI:** 10.1590/S1677-5538.IBJU.2017.0569

**Published:** 2018

**Authors:** Matheus N. Ribeiro da Silva, Aline Mendes, João R. Maciel Martins, Marcos Tobias-Machado, Maria Aparecida da Silva Pinhal

**Affiliations:** 1 Departamento de Bioquímica, Universidade Federal de São Paulo (UNIFESP), São Paulo, SP, Brasil;; 2 Departmento de Urologia Faculdade de Medicina do ABC, Santo André, SP, Brasil

**Keywords:** Biomarkers, Glycosaminoglycans, Prostatic Neoplasms

## Abstract

**Purpose:**

The present study evaluates chondroitin sulfate (CS) and heparan sulfate (HS) in the urine and hyaluronic acid (HA) in the plasma of patients with prostate cancer before and after treatment compared to a control group.

**Materials and Methods:**

Plasma samples were used for HA dosage and urine for quantification of CS and HS from forty-four cancer patients and fourteen controls. Clinical, laboratory and radiological information were correlated with glycosaminoglycan quantification by statistical analysis.

**Results:**

Serum HA was significantly increased in cancer patients (39.68 ± 30.00 ng/ mL) compared to control group (15.04 ± 7.11 ng/mL; p=0.004) and was further increased in high-risk prostate cancer patients when compared to lower risk patients (p = 0.0214). Also, surgically treated individuals had a significant decrease in seric levels of heparan sulfate after surgical treatment, 31.05 ± 21.01 μg/mL (before surgery) and 23.14 ± 11.1 μg/mL (after surgery; p=0.029). There was no difference in the urinary CS and HS between prostate cancer patients and control group. Urinary CS in cancer patients was 27.32 ± 25.99 μg/mg creatinine while in the men unaffected by cancer it was 31.37 ± 28.37 μg/mg creatinine (p=0.4768). Urinary HS was 39.58 ± 32.81 μg/ mg creatinine and 35.29 ± 28.11 μg/mg creatinine, respectively, in cancer patients and control group (p=0.6252).

**Conclusions:**

Serum HA may be a useful biomarker for the diagnosis and prognosis of prostate cancer. However, urinary CS and HS did not altered in the present evaluation. Further studies are necessary to confirm these preliminary findings.

## INTRODUCTION

Prostate cancer has a highly variable and unpredictable course. Currently, prostate cancer is diagnosed and its aggressiveness is classified by tumor stage, Gleason score, the extent of tumor at biopsy, and serum levels of prostate-specific antigen (sPSA). When relying solely on that information, patients are potentially unnecessarily over-diagnosed and thus sometimes over-treated, which has resulted in the 2012 U.S. Preventive Services Task Force recommendation of “D” (i.e. discouraged) for sPSA as a routine screening test. In 2017 the recommendation was updated and for men between 55 to 69 years the new recommendation is C, meaning that the decision about whether to be screened for prostate cancer should be an individual one. This discussion has increased the efforts to identify molecules that are expressed in prostate cancer and that can be associated with invasion and metastasis to improve the current prognostic capabilities and management of prostate cancer (1).

Changes in the levels and structure of glycosaminoglycan (GAG) side chains of proteoglycans have been associated with the development and progression of malignancies in various tissues (2-5). Likewise, prostate cancer has been shown to express GAGs, quantitatively and qualitatively, differently from normal and hyperplastic prostatic tissues. Moreover, the magnitude of this difference may even be of prognostic significance (6-13).

Heparan sulfate (HS) plays an important role in cell-cell and cell-matrix communication and cellular signaling, being an essential part of the cell microenvironment. It is extremely important in both development and cancer progression due to its regulation of cellular processes such as angiogenesis, tumor growth, proliferation, tumor invasion and metastasis. HS controls a variety of biological functions by modulating growth factor signaling pathways, such as FGF, VEGF and TGF- (14).

Heparan sulfate expression in prostate tumors is unlike normal human prostate tissue mainly due to decreased HS content in tissue stroma and heterogeneous HS expression in different tissue compartments (13). Overexpression of syndecan, a heparan sulfate proteoglycan, in prostate cancer was significantly associated with established features indicative of worse prognosis such as higher preoperative PSA, higher Gleason score, positive surgical margins, an extraprostatic extension of disease and biochemical disease progression. Also, metastatic prostate cancer tends to exhibit higher levels of both syndecan and perlecan, another heparan sulfate proteoglycan present at basement membrane (12).

The concentration of chondroitin sulfate (CS) is greatly increased over normal tissue levels in several different malignancies (4, 15), including prostate cancer. Indeed, elevated levels of sulfated chondroitin in the prostate peritumoral stroma are associated with higher incidence of PSA failure in radical prostatectomy (16). Moreover, chondroitin sulfate levels in advanced (cT4) prostate cancer tissues are very similar to the levels present in those early-stage prostate tumors that ultimately progressed (17).

Many tumor types have hyaluronan (HA) as a major part of the extracellular matrix (18). HA is a GAG important for cell division, cell migration and angiogenesis during embryogenesis, inflammation and wound healing (19). HA favors tumor cell invasion, epithelial to mesenchymal transition, cell proliferation, angiogenesis, lymphangiogenesis, and it recruits bone marrow-derived inflammatory and progenitor cells to tumors (18). In prostate cancer, accumulation of HA in tumor stroma and altered hyaluronic acid synthase and hyaluronidase in tumor epithelial cells are associated with increased cell proliferation, invasion, metastasis and poor outcome in men who have undergone radical prostatectomy (6, 7, 20-23).

The aforementioned studies found altered GAG expression in prostate tissue, mainly in the retrospective analysis of patients previously treated for the cancer, when the decision has already been made. However, the main objective of novel biomarkers is to help in the challenging decision-making of how to manage a patient recently diagnosed with prostate cancer. This study evaluates chondroitin sulfate and heparan sulfate in the urine and hyaluronan in the plasma of prostate cancer patients before and after treatment and correlated with known prognostic parameters. A comparative study was also performed with a control group, men that are unaffected by cancer.

## MATERIAL AND METHODS

We prospectively collected urine and blood from 44 men newly diagnosed with prostate cancer and 14 controls not eligible for prostate biopsy, considered low risk of harboring prostate cancer (PSA <1.5; non-suspicious digital rectal examination), according to the public academic urology service, during the year of 2009. Cancer patients were evaluated for serum PSA, Gleason grading performed according to the new modified system based on the 2005 consensus conference (24), D’Amico's clinical risk stratification, pelvic computed tomography and bone scan evaluations for distant metastasis. Cancer patients were treated with open or laparoscopic radical prostatectomy (32 patients), external beam radiotherapy (7 patients), and palliative hormone therapy for advanced disease (5 patients). Urine and blood samples were also collected after treatments, at 3, 6 and 12 months after. All work was performed with the institution-approved protocol with patient consent (ethical committee approval number CEP 019/2009).

Urinalysis and antibiogram were performed, as well as clinical evaluation of other urinary or systemic diseases such as urinary tract infection, functional bladder diseases, mucoviscidosis, diabetic renal disease and amyloidosis. If any of these diseases were present, the patient was excluded.

### 

#### Urinary Sulfated Glycosaminoglycan Quantification

Urine was collected from patients with prostate cancer and healthy subjects (about 50 mL). Urine samples were placed at 60°C for 1 hour for complete solubilization of proteins. Each sample was filtered using a paper filter at 4°C and the filtrate were centrifuged at 2,500 x g for 20 min. The supernatant (around 10 mL) was concentrated on Millipore filter with a 5,000 Da exclusion limit by centrifugation at 2,500 x g until achieving a total volume of 250 μL. The material (250 μL) was totally dried using vacuum and resuspended in distilled water to a final volume of 5 μL and subjected to agarose gel electrophoresis. Sulfated glycosaminoglycans (HS, DS and CS) were identified and quantified by agarose gel electrophoresis in 0.05 M 1,3-diaminopropane-acetate buffer (PDA), pH 9.0. After electrophoresis, for 1 h, at 100 V, the glycosaminoglycans were precipitated in agarose gel using 0.1% cetyltrimethylammonium bromide (CETAVLON), (Sigma-Aldrich, Saint Louis, MO) for 2 hours at room temperature. The gel was dried and stained with toluidine blue (0.1% in acetic acid: ethanol: water; 0.1 : 5 : 4.9, v:v:v). GAG quantification was carried out by densitometry at 530 nm. The extinction coefficients of the GAGs were calculated using standards of chondroitin 4-sulfate from whale cartilage (Seikagaku Kogyo Co., Tokyo, Japan), dermatan sulfate (from pig skin) and heparan sulfate (from bovine pancreas). The agarose gel electrophoresis method error was on the order of 5%.

#### Quantification of a non-sulphated glycosaminoglycan hyaluronic acid

HA was measured by a previously described fluorescence-based assay (25). Briefly, standard concentrations (0-500 mg of link protein) of HA obtained from human umbilical cord and urine samples from patients, diluted 1:4 in blocking buffer (100 μL of urine plus 300 μL of blocking buffer) were used. One hundred microliters from each solution was added, in triplicate, into the plates coated with hyaluronic acid-binding protein (HABP). The plates were incubated at 25°C, for 18 hours, washed three times with washing buffer and 100 mL of biotinylated HABP (1 mg/ mL), diluted in blocking buffer (1:5000) added to each well. The incubation was performed for 120 min. at 25°C under shaking. The plates were washed six times with washing buffer, and 100 μL of europium-labeled streptavidin (1:5000 in blocking buffer) were added. Incubation was carried out for 30 min. at 25°C, and washed six times to remove unbound streptavidin. Finally, 200 μL of enhancement solution (Perkin-Elmer Life Sciences-Wallac Oy, Turku, Finland) was added to release the europium bound to streptavidin and the plates were shaken for 10 min. A time-resolved fluorometer (Victor 2 from Perkin-Elmer, Life Sciences-Wallac Oy, Turku, Finland) was used to measure free europium and the fluorescence (counts/s). The values were processed automatically in the MultiCalc software program (Perkin-Elmer Life Sciences-Wallac Oy, Turku, Finland). This technique measures HA in concentration as low as 0.2 μg/L.

### Statistical analysis

The variables in the study were considered parametric or not based on the Kolmogorov-Smirnov test. Student t-test and ANOVA with Tukey's auxiliary test or Kruskal-Wallis and Mann-Whitney tests were used to compare parametric and non-parametric data, respectively. A significance level of 0.05 was adopted in all analysis. Statistical analysis was performed using SPSS version 23.0 (SPSS Inc., Illinois, USA).

## RESULTS

Table-1 summarizes clinical features such as serum PSA, Gleason score, D’Amico's clinical risk group and other clinical data from the patients as well as unaffected individuals.

**Table 1:** Clinical characteristics of cancer patients and control group.

	Cancer Patients	Control Group
**AGE**	68 years (46-77)	62 years (49-72)
**PSA**	27.48 ng/dL (1.4-150)	1.75 ng/dL (0.35 – 3.2)
**DRE**	Normal: n=18	Normal: n=14
	Nodule: n=23	
	diffusely endured prostate: n=3	
**Gleason (biopsy)**	4: n=1	
	6: n=17	
	7: n=15	
	8: n=5	
	9: n=6	
**Computed tomography**	Normal: n=41	
	Enlarged pelvic LN: n=3	
**Bone Scan**	Negative : n=43	
	Positive: n=1	
**D’Amico's clinical risk**	Low: n=15	
	Intermediate: n=8	
	High: n=21	

The numbers in parenthesis represents the average; **DRE** = Digital Rectum Examination; **n** = number of patients; **LN** = Lymph nodes

**Figure 1:**
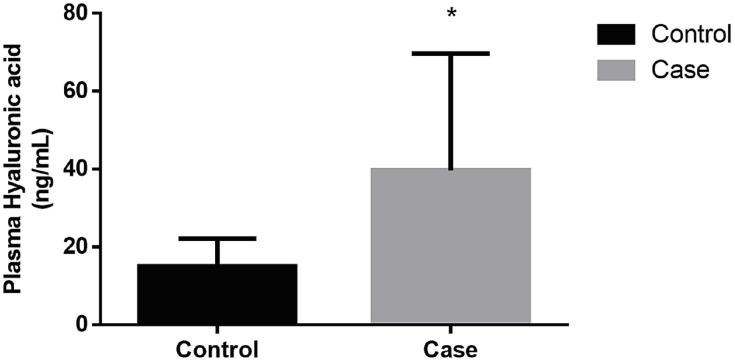
Profile of hyaluronic acid in cancer patients (Case) and individuals non affected by prostate cancer (Control). Hyaluronic acid was quantified in the plasma samples as previously described in Methods, using an ELISA-like assay. P = 0.004, Student-T Test.

**Figure 2:**
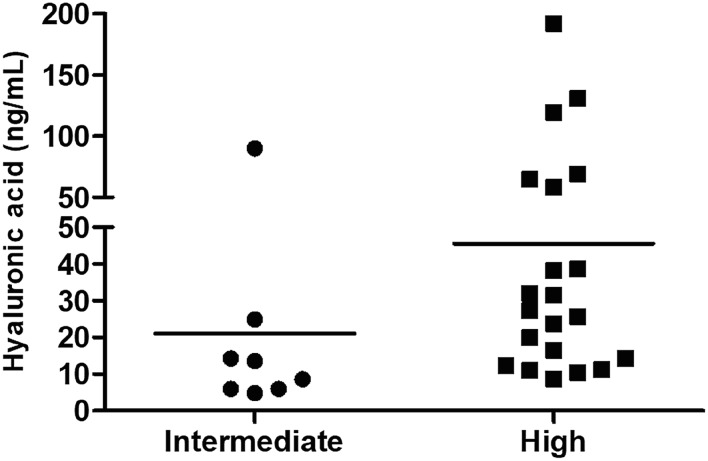
Hyaluronic acid in prostate cancer patients according to D'Amico's risk classification (Intermediate and High). There was a significant difference in the amount of plasma hyaluronic acid comparing the group of intermediate and high D'Amico's risk. p = 0.0214, Student-t Test.

Serum hyaluronic acid (HA) was significantly increased in cancer patients (39.68 ± 30.00 ng/mL) compared to the control group (15.04 ± 7.11 ng/mL); (p=0.004; Mann-Whitney test), as shown in Figure-1. Interestingly, HA was further increased in the group of patients that presented high-risk prostate cancer compared to intermediate risk patients (p = 0.0214; Mann-Whitney test), as shown in Figure-2. Patients with metastatic disease, positive bone scans or TC disclosing increased lymph nodes, have higher levels of hyaluronic acid (45.19 ± 7.32 ng/mL) compared to non-metastatic patients (15.16 ± 10.76 ng/mL), whereas significance was not achieved probably due to the small number of metastatic individuals (n=4; p=0.31; Mann-Whitney test).

There was no statistical difference in the urinary sulfated glycosaminoglycans (CS and HS) between prostate cancer patients and control group. In prostate cancer patients, the urinary CS was 27.32 ± 25.99 μg/mg creatinine and for unaffected individuals 31.37 ± 28.37 μg/mg creatinine (p=0.4768; Mann-Whitney test). There was also no significant difference in the amount of urinary heparan sulfate between patient and control groups, 39.58 ± 32.81 μg/mg creatinine and 35.29 ± 28.11 μg/mg creatinine, respectively (p=0.6252; Mann-Whitney test). Unlike hyaluronic acid urinary, chondroitin sulfate and heparan sulfate were not different for D’Amico's risk groups (p=0.471 and p=0.811 respectively; Kruskal Wallis test).

A significant increase in urinary chondroitin sulfate, was detected after hormone ablation compared to the data obtained before the treatment (41.01 ± 24.14 μg/mg creatinine and 24.14 ± 22.64 μg/mg creatinine, respectively) (Table-2). Therefore, patients with prostate cancer that had been submitted to hormone therapy presented higher levels of chondroitin sulfate compared to pre-treatment values. Conversely, surgical treatment promoted a significant decrease in the urinary level of heparan sulfate, 23.14 ± 11.1 μg/mg creatinine, compared to the result obtained before surgery, 31.05 ± 21.01 μg/mg creatinine, as shown in Table-2.

**Table 2:** Chondroitin sulfate and heparan sulfate quantified before and after treatment.

	Chondroitin Sulfate			Heparan Sulfate		
	(μg/mg creatinine)			(μg/mg creatinine)		
	Before	After treatment	P	Before	After treatment	P
Surgery	24.48 ± 21.25	21.98 ± 16.67	0.062	31.05 ± 21.01	23.14 ± 11.1	0.021
Hormone Therapy	24.14 ± 22.64	41.01 ± 24.14	0.042	39.12 ± 33.97	41.73 ± 21.60	0.71

p values based on Student-t test

When analyzing others laboratorial parameters, we observed some finds not considered in the initial hypothesis. Using the Spearman's rank correlation coefficient, there was an association between testosterone levels with chondroitin sulfate and heparan sulfate values secreted in the urine of patients with prostate cancer. The results show the higher the level of total testosterone, the higher the amount of the urinary chondroitin sulfate (p = 0.013) and heparan sulfate (p = 0.023). However, higher levels of free testosterone only revealed an increased amount in chondroitin sulfate (p = 0.019), with no significant alteration in heparan sulfate (p = 0.076), as demonstrated in Table-3.

**Table 3:** Evaluation of Gleason, PSA and the level of testosterone with the amount of sulfated glycosaminoglycans.

	Chondroitin Sulfate	Heparan Sulfate
	(μg/mg creatinine)	(μg/mg creatinine)
Gleason	p = 0.842	p = 0.675
Total PSA	p = 0.821	p = 0.993
Total Testosterone	p = 0.013	p = 0.023
Free Testosterone	p = 0.019	p = 0.076

The results were obtained in the group of patients before surgery and that were not submitted to hormone therapy; p values based on Spearman's rank correlation coefficient.

## DISCUSSION

Urological associations worldwide still recommend prostate cancer screening, though in a narrower population group, reflecting influence from the US Preventive Task Force recommendation in 2012, and its draft update in 2017. This lack of agreement reflects in part the difficulty to accurately identify the patient who should undergo a biopsy for suspicious significant prostate cancer. Diagnosis and treatment of indolent disease have led to unnecessary morbidity and mortality. Efforts have been made to identify molecular markers that function in promoting invasion/ metastasis and could be added as adjuncts to the current diagnostic tools. To date, this study is the first to evaluate a possible role of urinary GAG and plasmatic HA in aiding prostate cancer diagnostic and prognostic evaluation.

Our results demonstrated a significant increase in serum hyaluronic acid in prostate cancer patients, and this increase is even higher in those with high-risk disease. Moreover, this increase in HA tends to be even higher in metastatic patients. Gomez et al. showed that HA expression in the stroma of prostate cancer and surrounding tissue is higher the higher the PSA, Gleason score and clinical grading (6). In his study, it could predict biochemical recurrence after radical prostatectomy.

It has also been demonstrated in other previous studies of prostate cancer tissue analysis for HA. When we compared plasmatic HA concentration in the different clinical risk groups, the high risk groups had the higher measured plasmatic HA concentrations, which is consistent with previous immunohistochemical studies, where more aggressive cancer demonstrates higher HA expression in the prostate tissue (26, 27). If serum HA concentration is significantly increased in prostate cancer patients compared to non-cancer counterparts as demonstrated herein, it could be a useful tool to help identify patients at risk of harboring prostate cancer, either by adding PSA to routine screening or helping to select patients for re-biopsy. For instance, further study may indicate, in screening patients with low plasmatic HA levels, the likelihood of these patients developing prostate cancer. Likewise, in patients with rising PSA after a negative biopsy, if plasmatic levels of HA could help to determine which patient is at higher risk and therefore should undergo a new biopsy.

The small number of cases observed for metastasis contributes to the difficulty in making a strong association between these parameters and plasmatic HA. A larger series may help to confirm if metastatic patients do indeed have higher HA levels. An extended period of patient follow-up with data acquisition will further determine whether HA could also be an indicator of prostate cancer progression.

In our study, urinary measurement of CS and HS was similar in patients with and without prostate cancer, in contrast to a previous tissue study where both were increased in prostate cancer patients and also showed prognostic significance (8, 9, 16, 28, 29). In most studies, the main difference in tumoral CS concentration was structural, usually in the sulfation status, which could explain why it does not increase urinary CS. Likewise, the main studies where HS was increased in prostate cancer tissues was in its proteoglycan form and, therefore, possibly not increase HS urinary concentration (12, 30). However, we found a significant decrease in urinary HS after surgical extirpation of disease (and a decreasing trend in urinary CS), suggesting the possibility that a meaningful difference in sulfated urinary GAG concentration between cancer and non-cancer patients may be a matter of sample size.

Hormonal therapy resulted in an increase in urinary GAG concentrations, albeit in a small group. Also, there was a positive association between serum testosterone levels and urinary GAG concentration, pointing to a possible interference of steroid hormones in GAG synthesis.

As a preliminary study, the sample size is a major limitation to the strength of our conclusions, whereas for HA the difference between the groups was so striking that statistic tests provided strong results. Undoubtedly, validation of our finds in larger studies is essential to confirm HA as a useful biomarker and possibly encounter a role for the others studied GAGs, as suggested by others authors.

## CONCLUSIONS

In conclusion, we have shown a significant increase in serum hyaluronic acid in prostatic adenocarcinoma patients compared with controls, and such augment seems to be greater with higher grade of D’Amico's risk and metastatic patients. The results suggest that HA may be useful as a biomarker and predictive of disease aggressiveness in prostate cancer patients. However, a larger study is necessary to confirm these results, in order to define whether plasmatic HA measurement could be used to identify and help determine prognosis for prostate cancer patients, what would be particularly interesting since it is a non-invasive method.

## ABREVIATIONS

sPSA: = serum levels of prostate-specific antigen
GAG: = glycosaminoglycan
HS: = Heparan sulfate
CS: = chondroitin sulfate
HA: = hyaluronan or hyaluronic acid
